# An 11-Year-Old Male with Refractory Osteomyelitis

**DOI:** 10.1155/2012/285980

**Published:** 2012-12-03

**Authors:** Clifford T. Mauriello, Ole A. Raustol, Maria A. Aguiar, Kenji M. Cunnion

**Affiliations:** ^1^Department of Pediatrics, Eastern Virginia Medical School, 855 West Brambleton Avenue, Norfolk, VA 23510, USA; ^2^Children's Hospital of The King's Daughters, Children's Surgical Specialty Group, 601 Children's Lane, 855 West Brambleton Avenue, Norfolk, VA 23507, USA

## Abstract

We present a case of empirical treatment failure for chronic osteomyelitis in a previously healthy 11-year-old male involving the distal phalanx of the right first digit. After initial debridement, empiric antibiotics were started for presumed *Staphylococcus aureus* infection. Operative bacterial cultures yielded no growth. Despite three weeks of antistaphylococcal antibiotics the patient's symptoms worsened and the destruction of bone progressed. A repeat plain X-ray revealed a new lesion in the proximal phalanx of the right second digit. The recognition of multifocal osteomyelitis led to reexamination of bone tissue specimens using special stains which demonstrated rare broad-based budding yeast. Fungal cultures eventually grew *Blastomyces dermatitidis*. Treatment with amphotericin B led to rapid clinical improvement. This case illustrates that clinicians must remain vigilant for warning signs that empiric treatment may be failing for presumptive *Staphylococcus aureus*, provoking reconsideration of the differential diagnosis and an intensification of efforts to evaluate for alternative etiologies.

## 1. Case Presentation

An 11-year-old African American male with a history of nail biting presented to our hospital with a one-month history of painful swelling of his right thumb. The swelling appeared to be related to a small paronychia which the patient had been picking. Fever, systemic symptoms, and purulent discharge from the thumb were absent. He was evaluated by his primary care physician (PCP) at the beginning of the illness and treated with a course of amoxicillin-clavulanate. Over the next three weeks, his thumb became increasingly erythematous, edematous, and tender while taking antibiotics. He then returned to his PCP, who obtained a plain film that revealed cortical disruption at the base of the distal phalanx of the thumb. 

The child was then admitted to the hospital for osteomyelitis, and clindamycin was initiated for empiric treatment for presumed *Staphylococcus aureus *infection. A single-peripheral blood culture was obtained, which yielded no growth. A complete blood count, erythrocyte sedimentation rate, and C-reactive protein level were normal. Magnetic Resonance Imaging (MRI) of the right hand demonstrated abnormal signal in the distal phalanx and proximal phalanx of the thumb, including enhancement with intravenous gadolinium. 

The orthopedic surgeon performed open debridement of the infected bone. Intraoperatively, the distal phalanx was noted to have eroded into discrete halves. Purulent material was recovered from the pulp of the thumb and the bone. The wound was cultured for aerobic and anaerobic organisms and fungus, and bone specimens were sent for histopathology. H & E stains were performed on the tissue and showed reactive bony changes but were negative for granulomata. Thorough debridement and irrigation was performed. Clindamycin was continued postoperatively, and the patient appeared to improve initially. Operative aerobic and anaerobic cultures were negative for growth. The fungal culture was negative for growth at hospital discharge. A PICC line was placed for long-term IV therapy. A CXR was normal. A four- to six-week course of empiric intravenous clindamycin was planned.

**Figure 1:**
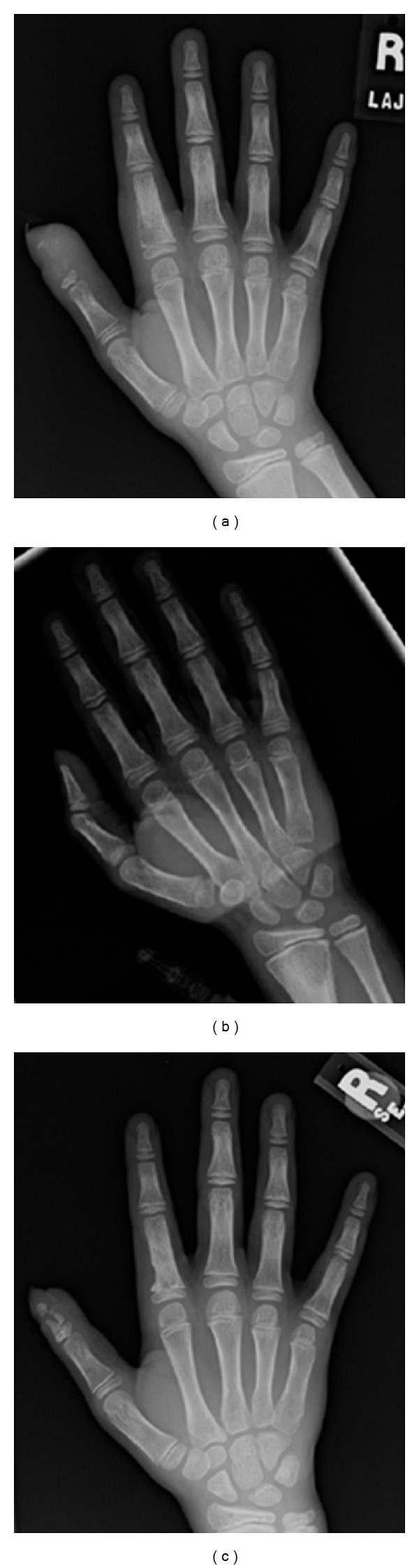
(a) Plain film of the right hand showing almost complete demineralization of the distal phalanx of the thumb and periosteal elevation and osteolysis of the proximal phalanx of the second digit. (b) Plain film obtained during skeletal survey after readmission, demonstrating an osteolytic lesion of the distal left radius. (c) Plain film of the right hand obtained after four months of antifungal therapy, demonstrating remineralization of the distal phalanx.

Three weeks following his discharge, he developed increased pain and swelling in his thumb. Additionally, a pustule had developed on the same thumb at a site distant from his surgical incision. Fever and systemic symptoms were absent. Purulent material was aspirated from the pustule in the office and sent for bacterial culture; this was also negative for growth. New plain films were obtained (Figure 1(a)) demonstrating nearly complete decalcification of the distal phalanx, as well as a new lucency in the proximal phalanx of the index finger. The child was readmitted, and his antibiotics were changed to vancomycin and cefazolin. 

The orthopedic surgeon performed a second debridement, revealing that most of the distal phalanx had been destroyed, but that the epiphysis was still viable. The thumb and index finger were debrided and irrigated. New aerobic and anaerobic operative bacterial cultures were taken, but yielded no growth. Three subsequent debridements were performed over the next week. Bacterial cultures were performed at each debridement, yet yielded no growth. Because lesions had been identified in two digits, a skeletal survey was performed, demonstrating a destructive lytic lesion of the left radius (Figure 1(b)). Due to progressive worsening of the infection despite aggressive management, the Infectious Diseases team raised concern that the presumptive diagnosis of *S. aureus* infection was likely incorrect. 

**Figure 2:**
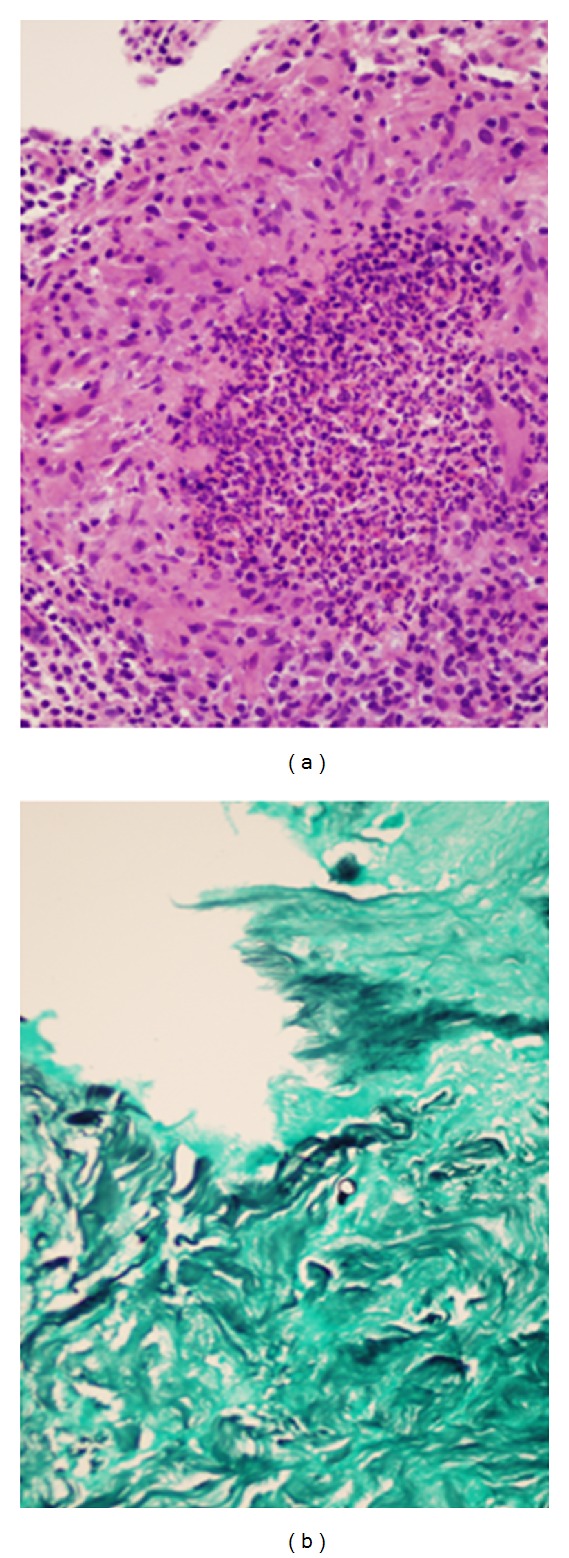
(a) Hematoxylin and eosin (H&E) stain from the second debridement demonstrating granulomatous inflammation. (b) Gomori methenamine silver (GMS) stain from the second debridement demonstrating broad-based budding yeast or “baby bottle” morphology of *Blastomyces dermatitidis*.

Five days after readmission, the orthopedic surgeon repeated a bone biopsy. Granulomas with neutrophilic centers were seen on H & E slides (Figure 2(a)). At the request of the Infectious Diseases team, the biopsy specimens were processed for special stains. GMS stains of this specimen demonstrated broad-based budding yeast suggestive of blastomycosis (Figure 2(b)). Silver stains were then performed on the first biopsy, and these were also positive for broad-based budding yeast. The fungal culture from the patient's first operation was then reexamined, demonstrating mold, thus a thermally dimorphic fungus. Conidial morphology under microscopy was consistent with *Blastomyces dermatitidis*. Vancomycin and cefazolin were discontinued. The results of the culture and the biopsy were confirmed by a commercial reference laboratory (Quest Diagnostics) via polymerase chain reaction and DNA probe. Antifungal therapy was initiated with amphotericin B deoxycholate and subsequently switched to liposomal amphotericin B to complete a 14-day course. 

The patient was discharged home on oral itraconazole for an anticipated one-year course. Following four months of therapy, his distal phalanx had significantly remineralized (Figure 1(c)). His wound healed completely.

## 2. Discussion

*Staphylococcus aureus* remains the most common cause of osteomyelitis [1–4], and because this patient's presentation was associated with a paronychia, empiric therapy was directed against this organism. Empiric treatment for *S. aureus* is a commonly used approach when no microbiological data is available or cultures yield no growth. Although empiric treatment with antistaphylococcal antibiotics frequently is associated with a favorable clinical outcome, it is vital that clinicians remain vigilant that, in the absence of microbiological proof of etiology, empirical therapy may be incorrect. In the field of quality improvement, this problem is sometimes referred to as “undue certitude,” which can significantly delay the time to initiation of effective therapy. This child's course is illustrative of how delay in seeking an alternative microbiological diagnosis can result in progressive tissue destruction. The ability to recognize and respond to warning signs that empirical therapy is failing is of utmost importance. In this patient's case, there were several important warning signs that empirical therapy was failing, suggesting that the presumed etiology was incorrect. The differential diagnosis was reconsidered and alternate microbiological diagnoses were sought, ultimately resulting in a favorable outcome. 

The recognition of multifocal lytic bone lesions was also a critical feature that prompted reconsideration of the differential diagnosis. At this age, it would be extremely unusual for *Staphylococcus aureus* to cause multifocal osteomyelitis, but this can be seen with *Mycobacterium tuberculosis, *nontuberculous* Mycobacterium, Bartonella henslase, Brucella *species*, Coxiella burnetti, *yeast, mold, and the dimorphic fungi. The differential diagnosis also includes chronic recurrent multifocal osteomyelitis, Synovitis Acne Pustulosis Hyperostosis Osteitis Syndrome, histiocytosis, Ewing's sarcoma, and leukemia.

The patient's localized signs and symptoms worsened after optimal open debridement and empiric antibiotic therapy for *Staphylococcus aureus, *suggesting that his IV antibiotic regimen was failing. Thus, either a clindamycin-resistant *S. aureus* or some other microorganism was causative. Large amounts of purulent material were recovered from several open debridement procedures and sent for testing, yet multiple Gram stains and all of his bacterial cultures were negative for growth. Antibiotic failure and inability to recover bacteria from culture suggested that the presumptive diagnosis was incorrect and should be reconsidered.

Finally, our patient lacked a history of either traumatic injury or foreign body, which were the most common risk factors documented in two recent case series of bacterial chronic osteomyelitis [5, 6]. It is also important to note that he did not have sickle cell disease, vascular insufficiency, or neurological deficits that predispose to Gram-negative infections. 

Our patient's presentation has both typical and atypical features of the rare pediatric condition of disseminated *Blastomyces dermatitidis* infection. *Blastomyces dermatitidis* is a dimorphic mycosis which causes systemic pyogranulomatous disease. Mycelial conidia are acquired via the respiratory route [7]. In the lungs, the conidia convert into the yeast phase, escape containment by macrophages and neutrophils [8], and then disseminate throughout the body and stimulate granulomatous inflammation [9–11]. Bone and joint infections are the second most common extrapulmonary manifestation of blastomycosis after skin infections [10–13]. Well-circumscribed osteolytic lesions or destructive processes can be seen [14, 15]. The surrounding soft tissue is commonly involved as well and sinuses may be present [14, 15]. Children represent less than ten percent of all cases of blastomycosis [16]. In a recent case series, less than 50% of patients with *Blastomyces *osteoarticular infections had systemic symptoms and 59% had evidence of pulmonary pathology [15]. Osteomyelitis in the absence of lung findings has been previously described in older case series as well [14]. It is interesting to note that 19% of patients in the recent case series had multifocal osteomyelitis [15].

Histopathology and culture remain the gold standard for the diagnosis of disseminated blastomycosis. Blastomycosis forms are easily recognizable on silver stains, but there is typically a paucity of organism often requiring diligent search. The presence of broad budding yeast forms in tissue correlates well with culture results [17]. A blastomycosis urine antigen test has been shown to be very sensitive for severe and disseminated disease but is nonspecific due to cross reactivity with antigens from *Histoplasma capsulatam *[18, 19]. 

If possible, specimens for microbiological evaluation and histopathological evaluation should be obtained prior to initiation of antibiotic therapy. Due to the difficulty in obtaining these specimens, evaluation for bacterial, fungal, and mycobacterial organisms is warranted. Appropriate testing of specimens can be facilitated by early consultation with infectious disease experts.
